# DIet and Health From reGIstered Trials on ClinicalTrials.gov: The DIGIT Study

**DOI:** 10.3389/fnut.2022.870776

**Published:** 2022-04-25

**Authors:** Monica Dinu, Giuditta Pagliai, Cristian Del Bo', Marisa Porrini, Patrizia Riso, Mauro Serafini, Francesco Sofi, Daniela Martini, Donato Angelino

**Affiliations:** ^1^Department of Experimental and Clinical Medicine, University of Florence, Florence, Italy; ^2^Department of Food, Environmental and Nutritional Sciences (DeFENS), University of Milan, Milan, Italy; ^3^Faculty of Bioscience and Technology for Food, Agriculture and Environment, University of Teramo, Teramo, Italy

**Keywords:** Mediterranean diet, clinical trials, dietary intervention study, humans, diet, dietary pattern, low-carb diets, food timing

## Abstract

**Background:**

Clinical trial registration has become a valuable tool that can be used to track the status and nature of trials conducted on a specific topic. This approach has been applied to many areas of research, but less is known about the characteristics and trends over time of clinical trials focused on diet and health. The aim of this study was to analyze diet-related clinical trials registered on the National Institute of Health “ClinicalTrials.gov” web platform in the last 10 years, to list and describe their characteristics, and to identify possible gaps to be filled in the future research.

**Methods:**

A search was performed on the ClinicalTrials.gov database. Intervention studies registered from January 2010 to December 2020, conducted on adults, with a follow-up of ≥2 weeks, evaluating the impact of different diets on all outcomes except those assessed with scales or questionnaires were considered.

**Results:**

At the end of the selection process, a total of 1,016 registered clinical trials were identified and included in the analysis. The most investigated dietary approaches were balanced diets (*n* = 381 trials), followed by those based on a modification of macronutrients (*n* = 288) and time-restricted feeding and intermittent fasting diets (*n* = 140). The main measured outcomes included anthropometric parameters and body composition (57.8%), glycemic control parameters (49.7%), lipid parameters (40.1%), inflammatory markers (29.1%), and blood pressure and/or heart rate (24.5%). A growing body of studies also focused on microbiota and host metabolism (17.8%). Most studies had a duration of less than 12 weeks (~60%), and more than 90% of studies enrolled volunteers with overweight/obesity or other diseases. Regarding aging, only 21 studies focused only on older adults.

**Conclusion:**

The number of studies investigating the relationship between diet and health has increased over the years. Despite the growing interest in the topic, there are some gaps, such as the limited duration of most trials, the underrepresentation of some population groups, and the limited number of studies for some diets that, although popular in the population, lack robust scientific evidence.

## Introduction

The paramount importance of diet on human health is supported by a large body of scientific evidence. The Global Burden of Disease highlighted that, in 2017, 11 million deaths and 255 million disability-adjusted life years (DALYs) were attributable to dietary risk factors (1), supporting the need for improving dietary habits across countries.

The evaluation of the role of diet on human health has been the object of an enormous number of publications, and the interest in this research area is continuously growing. For instance, using “diet” as a search term in PubMed, 200,599 manuscripts resulted to be published before 2000, but the number grew to 319,908 in 2010 and is currently (December 30, 2021) 577,014. A large number of epidemiological and clinical evidence for health promotion is available for “traditional” diets, such as the Mediterranean diet or for low-carbohydrate diets (2). However, in the past years, besides these, other popular diets (e.g., ketogenic diet and intermittent-energy restriction) are gaining more interest for their potential effect on body weight and other health markers (2).

The growing number of publications on this topic suggests that many other manuscripts on diet and health will be published in the next years. However, it is well-known that manuscripts can be published many years after the completion of a study. In this sense, an alternative way to have more updated results about the research currently performed is to explore the registers of clinical trials. This allows to study the characteristics of past and present studies on diet to elucidate which are the most studied associations with health as well as to drive future research. This approach has been also used for other reviews, for investigating trials on specific topics (3–7), or for focusing on specific characteristics of the trials, such as sponsorship (8).

The World Medical Association stated in paragraph 35 of the Declaration of Helsinki that “*Every research study involving human subjects must be registered in a publicly accessible database before recruitment of the first subject”* (9). To have a shared and international platform, in 2004, there was a call to action of investigators asking to the World Health Organization for a public database for clinical trial registration. Then, the International Clinical Trials Registry Platform (ICTRP) was set-up, and “*the main aim of the WHO ICTRP is to facilitate the prospective registration of the WHO Trial Registration Data Set on all clinical trials and the public accessibility of that information*” (10). ClinicalTrials.gov is one of the largest registers, owning almost half of the total ICTRP-registered studies, and is maintained by the National Library of Medicine (NLM) at the National Institutes of Health (NIH) of the United States (11).

The aim of this study was to analyze all registered clinical trials having diet as the object of research, by retrieving information from ClinicalTrials.gov. This approach can offer a glimpse into the research pipeline of universities and research institutions, allowing us to group and identify fields with increased research that may be of clinical significance in the next decade.

## Materials and Methods

### Search Strategy and Study Selection

To identify studies focused on diet, a search was performed on the ClinicalTrial.gov database, using a combination of the following search terms: “diet” OR “dietary intervention.” Inclusion criteria were as follows: 1) Population: adults (aged 18–64 years) and/or older adults (>65 years); 2) study type: interventional studies with a follow-up of ≥2 weeks; 3) type of intervention: diets as a whole (no studies evaluating the effect of a single nutrient or food); 4) outcome: all outcomes except those assessed with scales or questionnaires; 5) status: all except “withdrawn”; and 6) registration date: all the trials registered from 1 January 2010 to 31 December 2020. The search was performed on 13 April 2021.

The study selection and the evaluation of the eligibility of the clinical trials were performed by two independent reviewers (D.M. and M.D.). Any discrepancy between reviewers was solved through consultation with a third independent author (G.P.) to achieve a consensus.

### Data Extraction

The following data were extracted from an XML dataset downloaded from the US National Library database “ClinicalTrial.gov” in “All Available Columns” and “Tab-separated values”: URL of the registered trial, title, year of first publication in the “ClinicalTrials.gov” register, study location, gender, age group, health status of study participants, number of enrolled subjects, type of diet, outcomes of interest, duration of the intervention, and funding sources. Data extraction from the registry was performed by two reviewers (MD and GP). A third author (DA) checked the extracted information to ensure the accuracy of the retrieved data.

The year of first publication on “ClinicalTrials.gov” was extracted to explore temporal trends. The health status of enrolled participants was assessed by considering the field *Conditions*. Trials stating that included participants were “healthy” were categorized as studies conducted on a healthy population. The other clinical conditions were analyzed by performing frequency statistics and were manually classified as follows: 1) overweight/obesity, 2) diabetes, 3) metabolic syndrome, 4) cardiovascular disease/risk, 5) cancer (all sites), 6) gastrointestinal condition, 7) liver disease, 8) kidney disease, 9) neurodegenerative disease, 10) polycystic ovary syndrome, and 11) other (e.g., rheumatoid arthritis, spinal cord injury, fibromyalgia, psoriasis, and HIV).

The type of diet being studied was extracted from the *Intervention field*. As a first step, the 10 most used diets in clinical trials, defined as “top 10 diets,” were analyzed. The number of studies (in absolute value) in which these diets were evaluated, the relative value per year, and the number and type of outcomes considered were reported. Then, to facilitate the organization and presentation of data, the diets were manually grouped into eight categories, termed “dietary approaches,” that shared common characteristics: 1) balanced diets, 2) diets based on the modification of macronutrients, 3) diets based on food timing, 4) diets that excluded specific foods or food groups, 5) diets based on the modification of specific nutrients/nonnutrients, 6) diets with meal replacement, 7) diets fortified with specific foods, and 8) other. Table 1 shows the diets that make up each of these categories. It should be noted that the categories were not mutually exclusive (e.g., when a balanced diet was compared with a diet based on macronutrient modification); therefore, trials were labeled with as many categories as were relevant. Consequently, the percent of trials by dietary approach sums to greater than 100%.

**Table 1:** Diets object of study in health-related trials registered on ClinicalTrials.gov from 2010 to 2020.

**Diet category**	**Number of diets**
Balanced diet (*n* = 381)	low-calorie balanced diet (*n* = 123), Mediterranean diet (n=106), diet based on official dietary guidelines (*n* = 39), DASH (*n* = 36), low-glycemic index diet (*n* = 36), Nordic diet (*n* = 14), DPP diet (*n* = 9), anti-inflammatory diet (*n* = 8), Weight Watchers diet (*n* = 7), traditional diet (*n* = 5), MIND diet (*n* = 2), portion controlled (*n* = 2), Nutritarian diet (*n* = 1), prudent diet (*n* = 1)
Modification of macronutrients (*n* = 288)	low-carbohydrate diet (*n* = 80), ketogenic diet (*n* = 69), high-protein diet (*n* = 58), low-fat diet (*n* = 40), high-PUFA diet (*n* = 15), high-MUFA diet (*n* = 13), low-protein diet (*n* = 13), high-fat diet (*n* = 10), high-carbohydrate diet (*n* = 8), high-SFA diet (*n* = 7), modified-Atkins (*n* = 5), low-SFA diet (*n* = 2)
Food timing (*n* = 140)	time-restricted feeding (*n* = 51), intermittent-fasting (*n* = 39), intermittent-energy restriction (*n* = 21), alternate-day fasting (*n* = 10), frequency of meals (*n* = 9), meal timing (*n* = 7), chronotype-adapted diet (*n* = 2), Ramadan (*n* = 2)
Exclusion of foods/food groups (*n* = 111)	vegetarian diet (*n* = 28), vegan diet (*n* = 23), low FODMAP diet (*n* = 23), gluten-free diet (*n* = 14), Paleo diet (*n* = 8), Portfolio diet (*n* = 6), exclusion of sugar (*n* = 4), Crohn's disease exclusion diet (*n* = 2), pesco-vegetarian diet (*n* = 2), exclusion of milk and cheese (*n* = 1), ulcerative colitis diet (*n* = 1)
Modification of nutrients/non-nutrients* intake (*n* = 72)	sodium (*n* = 23), fiber (*n* = 20), minerals (*n* = 7), resistant starch (*n* = 5), additives (*n* = 4), phenolic compounds (*n* = 4), amino acids (*n* = 3), choline (*n* = 2), fructose (*n* = 2), folate (*n* =1), oxalate (*n* = 1)
Meal replacement (*n* = 45)	fasting-mimicking diet (*n* = 17), meal replacement (*n* = 6), Medifast (*n* = 6), Nutrisystem-D (*n* = 4), Optifast (*n* = 4), Herbalife (*n* = 2), Modifast (*n* = 2), Colorado diet (*n* = 1), LOGI diet (*n* = 1), My New Weigh (*n* = 1), Phyto-Pro (*n* = 1)
Fortification with specific foods (*n* = 43)	whole grains (*n* = 7), meat (*n* = 7), fish (*n* = 4), avocado (*n* = 3), nuts (*n* = 3), pulses (*n* = 3), almonds (*n* = 2), flaxseeds (*n* = 2), legumes (*n* = 2), cereals containing gluten (*n* = 1), pistachios (*n* = 1)
Other (*n* = 29)	low-AGE diet (*n* = 6), western diet (*n* = 5), hypercaloric diet (*n* = 4), organic diet (*n* = 3), microbiome diet (*n* = 3), algorithm-based diet (*n* = 2), DNA-diet (*n* = 1), Gracie diet (*n* = 1), low-antigen diet (*n* = 1), Mito-Food plan (*n* = 1), nutrients sequence in main meals (*n* = 1)

*DASH, Dietary Approaches to Stop Hypertension; DPP, Diabetes Prevention Program; MIND, Mediterranean-DASH Intervention for Neurodegenerative Delay; PUFA, polyunsaturated fatty acids; MUFA, monounsaturated fatty acids; SFA, saturated fatty acids; FODMAP, fermentable oligosaccharides, disaccharides, monosaccharides, and polyols; AGE, advanced glycation end products*.

* *By definition, nonnutrients are substances found in food that can potentially affect human health but are not identified as nutrients, such as many bioactive compounds like polyphenols or carotenoids*.

Regarding outcomes, data were extracted from *Primary and Secondary outcomes* fields. Also in this case, the following categorization of outcomes was manually performed: 1) anthropometric parameters and body composition, 2) hemochrome parameters, 3) lipid parameters, 4) glycemic control parameters, 5) markers of liver function, 6) markers of kidney function, 7) markers of thyroid function, 8) minerals and vitamins, 9) hormonal parameters, 10) blood pressure and/or heart rate, 11) inflammatory markers, 12) oxidative stress parameters, 13) cancer biomarkers, 14) microbiota and metabolites, 15) genetic markers, and 16) other (e.g., brain perfusion or metabolites, bone metabolism density, and ejection fraction). Also in this case, trials assessing multiple outcomes were labeled with as many categories as were relevant, and consequently, the percentages of trials by outcome sum to greater than 100%.

The age of the participants was taken from the *Age* field and classified following the categorization in “ClinicalTrials.gov”: adults only (18–64 years), older adults only (≥65 years), and both adults and older adults (>18 years). As to the number of enrolled volunteers, data were extracted from the *Enrollment* field and categorized into 4 groups, namely, 1–50 participants, 51–100 participants, 101–500 participants, and >500 participants. The duration of the intervention was extracted from the *Duration* field and was categorized into ≤ 12 weeks, 13–24 weeks, 25–52 weeks, and >52 weeks. The funding source was categorized into Academic Medical Centers/Hospitals/United States Government/Others (AMC/Hosp/US Govt/Others), industry, and both. The category “US Govt” was generated from the “ClinicalTrials.gov” database categories NIH and US Fed, while “Other” was composed of charities and foundations. The category “Industry” was taken directly from the categorization in ClinicalTrials.gov.

### Data Analysis

Descriptive analyses were performed using the statistical package IBM Statistical Package for Social Science for Macintosh version 27.0 (SPSS 27.0, Armonk, NY, USA: IBM Corp.). In particular, frequency statistics were performed, and data were expressed as numbers and percentages of the total. Given the lack of specific reporting guidelines developed for this type of study registry analysis, we adhered to the Strengthening the Reporting of Observational Studies in Epidemiology (STROBE) reporting guidelines for cross-sectional studies (12).

## Results

### Selection and Characteristics of Studies

**Figure 1:**
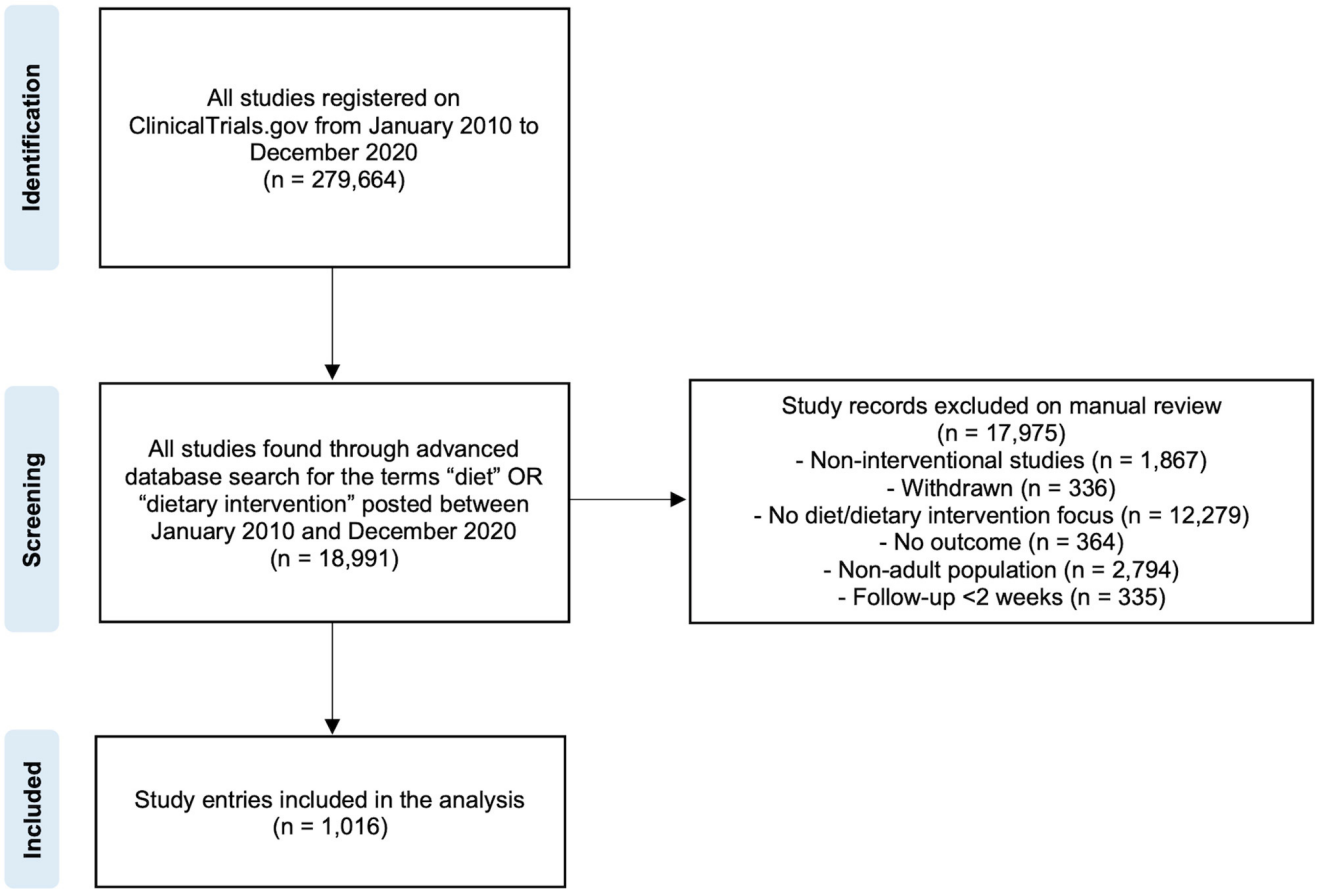
Flowchart for the selection procedure of diet- and health-related clinical study entries considered for the analysis.

A total of 18,991 diet-related studies were found in the ClinicalTrials.gov database by using the search strategy (Figure 1). Of these, 17,975 were removed because of not matching the inclusion criteria. At the end of the selection process, a total of 1,016 registered clinical trials were selected and included in the analysis.

Overall, 15.4% of the trials (*n* = 156) were conducted on women, 5.1% (*n* = 52) on men, and 79.5% (*n* = 808) on both sexes. In terms of age groups, 70.2% of the trials (*n* = 713) included both adults (18–65 years) and older adults (>65 years), while almost one-third were conducted in adults only (*n* = 283; 27.9%), and 2% (n=29) were conducted in older adults only. Almost half of the trials enrolled ≤ 50 participants (*n* = 460; 45.2%), while 27.6% (*n* = 280) and 24.8% (*n* = 252) of the studies included 51–100 and 101–500 participants, respectively. Only 2.4% (*n* = 24) of the trials enrolled >500 participants. Regarding the duration of the intervention, 54.8% of the trials (*n* = 557) had a follow-up of ≤ 12 weeks, in 23.8% (*n* = 242) ranged from 13 to 24 weeks, and in 13.2% (*n* = 134) ranged from 25 to 52 weeks, while 8.1% (*n* = 82) had a duration of more than 1 year. The study duration was not specified in 1 trial (0.1%).

With regard to the health status of the enrolled participants, they were defined as “healthy” in the 6.8% of the trials (*n* = 69), while 91.5% of the trials enrolled volunteers with diseases (i.e., overweight/obesity (*n* = 277), diabetes (*n* = 161), cardiovascular disease/risk (*n* = 100), cancer (*n* = 70), metabolic syndrome (*n* = 53), gastrointestinal conditions (*n* = 50), liver disease (*n* = 44), kidney disease (*n* = 32), neurodegenerative disease (*n* = 24), polycystic ovary syndrome (*n* = 15), and other (*n* = 104). In 17 trials (1.7%), participants' health condition was not specified. The analysis of funding sources showed that 91.3% of trials (*n* = 928) were funded by academic medical centers, hospitals, government agencies, or others, 1.2% (*n* = 12) by industry, and 7.5% (*n* = 76) by both.

### Registration of Trials Over Time, Recruiting Countries, and Outcomes Examined

**Figure 2:**
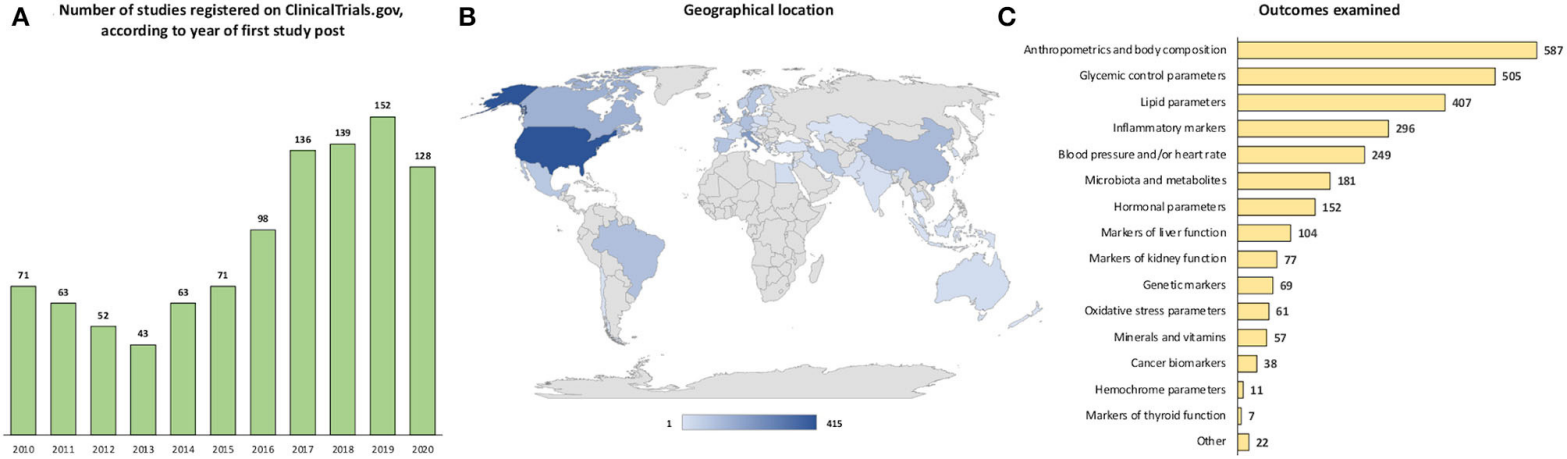
Number of studies registered on ClinicalTrials.gov, according to **(A)** year of first study post, **(B)** geographical location, and **(C)** examined outcome.

By considering the year of first publication in the “ClinicalTrials.gov” database, a continuous rise in diet- and health-related study entries has been observed since 2014, with the highest number of publications (*n* = 152) in 2019 and a subsequent slight decrease in 2020 (Figure 2A). Considering the geographical area of the trials (Figure 2B), more than one-third of the trials were located in the United States (*n* = 415; 40.8%), followed by Italy (*n* = 68; 6.7%), Canada (*n* = 47; 4.6%), the United Kingdom (*n* = 41; 4.0%), China (*n* = 39; 3.8%), Denmark (*n* = 37; 3.6%), Germany (*n* = 33; 3.2%), Brazil (*n* = 32; 3.1%), Spain (*n* = 31; 3.1%), Sweden (*n* = 29; 2.9%), Israel (*n* = 27; 2.6%), Mexico (*n* = 25; 2.5%), Iran (*n* = 23; 2.23%), Norway (*n* = 20; 2%), and the Netherlands (*n* = 18; 1.8%). The other countries shown in the map reported less than 10 eligible trials registered on “ClinicalTrials.gov” over the past decade.

As to the examined outcomes (Figure 2C), anthropometric parameters and body composition were the ones assessed in the largest number of studies (57.8%), followed by glycemic control parameters (49.7%), lipid parameters (40.1%), inflammatory markers (29.1%), blood pressure and/or heart rate (24.5%), microbiota and metabolites (17.8%), hormonal parameters (15%), markers of liver function (10.2%), markers of kidney function (7.6%), genetic markers (6.8%), oxidative stress parameters (6%), minerals and vitamins (5.6%), cancer biomarkers (3.7%), hemochrome parameters (1.1%), markers of thyroid function (0.7%), and other (2.2%).

### Top 10 Diets

**Figure 3:**
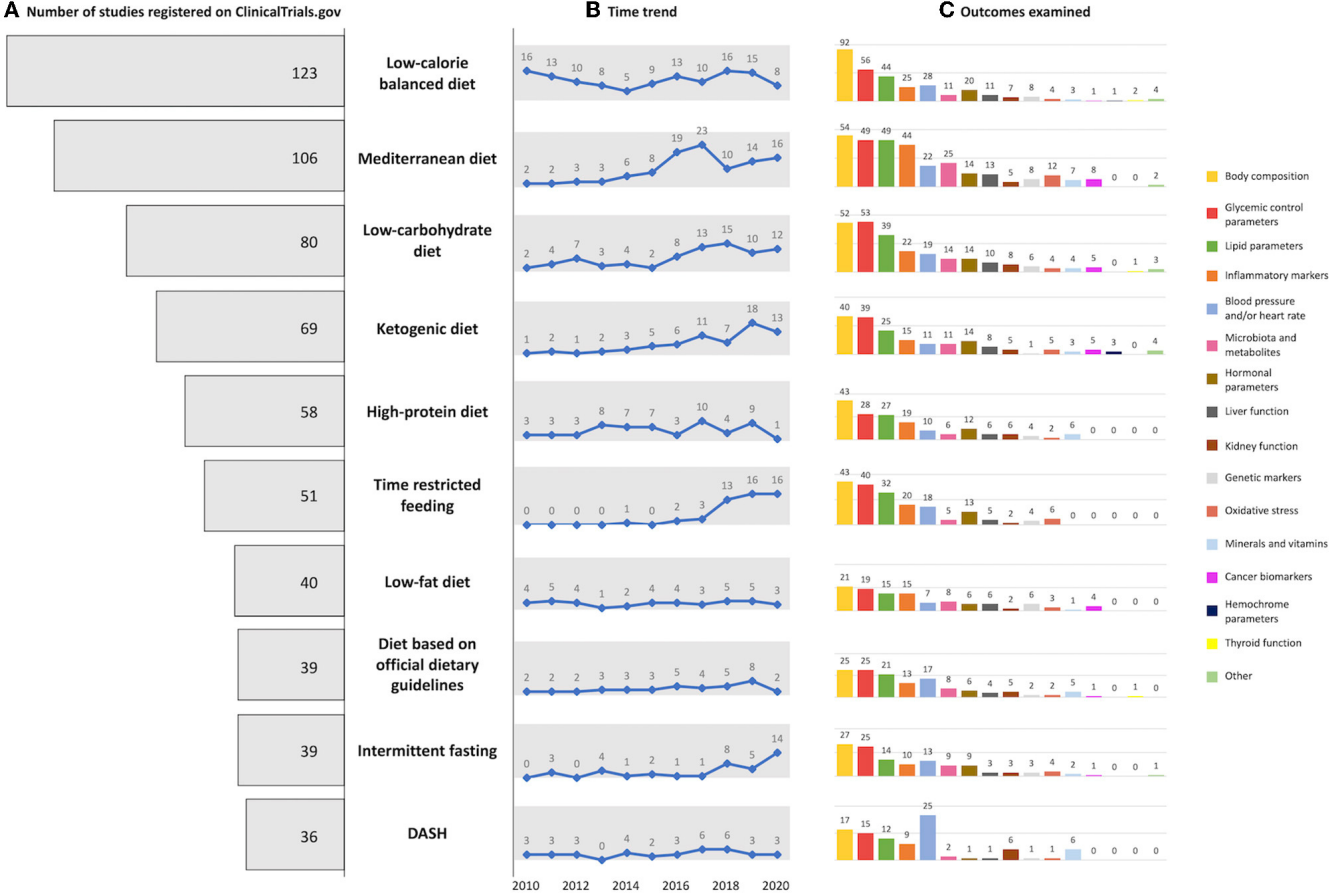
Graphical representation of **(A)** number of top 10 diets registered on ClinicalTrials.gov, **(B)** with the related time trend, and **(C)** examined outcome.

The top 10 diets used in clinical trials registered on “ClinicalTrials.gov” between 2010 and 2020 are reported in Figure 3. Of these, balanced low-calorie diets and the Mediterranean diet were the most highly rated, with 123 and 106 trials, respectively. By considering the time trend, Mediterranean diet, low-carbohydrate diet, ketogenic diet, time-restricted eating, and intermittent fasting were the fastest growing in the last years. Regarding outcomes, a similar trend was observed for all diets, with anthropometric measure being the mostly measure examined for all diets except for the low-carb diet for which glycemic control parameters prevailed.

### Dietary Approaches

By considering the dietary approach used in the registered trials, balanced diets accounted for the largest proportion of trials (37.5%), followed by diets based on the modification of macronutrients (28.3%), diets based on food timing (13.8%), diets that excluded specific foods or food groups (10.9%), diets based on the modification of specific nutrients/non-nutrient intake (7.1%), diets with meal replacement (4.4%), diets added with specific foods (4.2%), and other diets (2.9%) (Table 1).

Comparing the different dietary approaches (Table 2), studies focusing on macronutrient modification were performed mostly in men than women, while the number of studies focusing only on older adults was small for all the dietary approaches. In terms of the sample size and study duration, trials evaluating balanced diets reported on average more participants and longer follow-up. In contrast, about half of the registered trials focusing on the other diets involved the recruitment of fewer than 50 volunteers and had a follow-up of ≤ 3 months. In most cases, trials enrolled also subjects with overweight/obesity status or individuals with cardiometabolic disorder conditions. It is noteworthy that a large proportion of trials involving the exclusion of foods/food groups were conducted in subjects with renal disease (31.4%), while 22.7% of studies involving nutrient/nonnutrient modification were conducted in subjects with cancer. Regarding microbiota and derived metabolites as study outcomes, the analysis was found mainly in studies reporting the exclusion of foods/food groups, with over 40% of registered trials involving the assessment of these parameters. Finally, the industry appears to play a limited role in terms of funding, except for studies with meal replacements where nearly 50% of registered trials had full or partial private funding.

**Table 2:** Characteristics of included trials registered on ClinicalTrials.gov from 2010 to 2020 according to dietary approaches.

	**Balanced diet**	**Modification of macronutrients**	**Food timing**	**Exclusion of foods/food groups**	**Modification of nutrients/ non-nutrients**	**Meal replacement**	**Fortification with specific foods**	**Other**
**Gender**								
Women only	79 (20.7)	35 (12.2)	26 (18.6)	8 (7.2)	7 (9.7)	6 (13.3)	7 (16.3)	2 (6.9)
Men only	10 (2.6)	26 (9.0)	9 (6.4)	3 (2.7)	-	2 (4.4)	2 (4.7)	2 (6.9)
Both	292 (76.6)	227 (78.8)	105 (75.0)	100 (90.1)	65 (90.3)	37 (82.2)	34 (79.1)	25 (86.2)
**Age group**								
Adults only (18-65 years)	107 (28.1)	83 (28.8)	50 (35.7)	28 (25.2)	13 (18.1)	6 (13.3)	17 (39.5)	9 (31.0)
Older adults only (>65 years)	7 (1.8)	8 (2.8)	1 (0.7)	1 (0.9)	-	2 (4.4)	-	2 (6.9)
Both	267 (70.1)	197 (68.4)	89 (63.6)	82 (73.9)	59 (81.9)	37 (82.8)	26 (60.5)	18 (62.1)
**Number of enrolled participants**								
1–50	128 (33.6)	146 (50.7)	72 (51.4)	61 (55.0)	35 (48.6)	18 (40.0)	17 (39.5)	14 (48.3)
51–100	111 (29.1)	71 (24.7)	44 (31.4)	28 (25.2)	16 (22.2)	18 (40.0)	15 (34.9)	7 (24.1)
101–500	125 (32.8)	63 (21.9)	24 (17.1)	21 (18.9)	20 (27.8)	9 (20.0)	11 (25.6)	8 (27.6)
>500	17 (4.5)	8 (2.8)	-	1 (0.9)	1 (1.4)	-	-	-
**Duration of the intervention**								
≤ 12 weeks	170 (44.6)	165 (57.3)	95 (67.9)	68 (61.3)	48 (66.7)	18 (40.0)	25 (58.1)	21 (72.4)
13–24 weeks	101 (26.5)	66 (22.9)	26 (18.6)	28 (25.2)	9 (12.5)	13 (28.9)	12 (27.9)	4 (13.8)
25–52 weeks	67 (17.6)	31 (10.8)	15 (10.7)	10 (9.0)	10 (13.9)	8 (17.8)	2 (4.7)	3 (10.3)
>52 weeks	43 (11.3)	25 (8.7)	4 (2.9)	5 (4.5)	5 (6.9)	6 (13.3)	4 (9.3)	1 (3.4)
Not specified	-	1 (0.3)	-	-	-	-	-	-
**Health status of participants**								
Healthy population	12 (3.1)	21 (7.3)	16 (11.4)	6 (5.4)	6 (8.3)	-	8 (18.6)	6 (20.7)
Population with diseases								
Overweight/obesity	116 (31.7)	71 (27.3)	45 (37.2)	13 (12.7)	10 (15.2)	22 (50.0)	16 (37.2)	6 (26.1)
Diabetes	66 (18)	47 (18.1)	29 (24.0)	18 (17.6)	8 (12.1)	4 (9.1)	3 (7.0)	5 (21.7)
Cardiovascular disease/risk	14 (3.8)	19 (7.3)	9 (7.4)	4 (3.9)	4 (6.1)	1 (2.3)	2 (4.7)	3 (13.0)
Cancer	52 (14.2)	25 (9.6)	5 (4.1)	7 (6.9)	15 (22.7)	3 (6.8)	7 (16.3)	1 (4.3)
Metabolic syndrome	20 (5.5)	28 (10.8)	8 (6.6)	4 (3.9)	4 (6.1)	8 (18.2)	2 (4.7)	-
Gastrointestinal conditions	20 (5.5)	14 (5.4)	7 (5.8)	4 (3.9)	1 (1.5)	2 (4.5)	-	-
Liver disease	11 (3)	7 (2.7)	2 (1.9)	1 (1.0)	-	1 (2.3)	1 (2.3)	3 (13.0)
Kidney disease	10 (2.7)	3 (1.2)	2 (1.9)	32 (31.4)	4 (6.1)	1 (2.3)	2 (4.7)	1 (4.3)
Neurodegenerative disease	9 (2.5)	12 (4.6)	2 (1.9)	2 (2.0)	4 (6.1)	2 (4.5)	-	2 (8.7)
Polycystic ovary syndrome	7 (1.9)	4 (1.5)	4 (2.9)	2 (2.0)	-	-	1 (2.3)	-
Other	41 (11.2)	10 (11.5)	8 (5.7)	15 (14.7)	16 (24.2)	-	-	2 (8.7)
Not specified	3 (0.8)	7 (2.4)	3 (2.1)	3 (2.7)	-	1 (2.2)	1 (2.3)	
**Outcomes**								
Anthropometrics/body composition	243 (63.8)	162 (56.3)	108 (77.1)	49 (44.1)	27 (37.5)	25 (55.6)	20 (46.5)	13 (44.8)
Glycemic control parameters	196 (51.4)	151 (52.4)	100 (71.4)	44 (39.6)	23 (31.9)	19 (42.2)	20 (46.5)	9 (31.0)
Lipid parameters	153 (40.2)	127 (44.1)	73 (52.1)	40 (36.0)	14 (19.4)	13 (28.9)	25 (58.1)	5 (17.2)
Inflammatory markers	113 (29.7)	82 (28.5)	39 (27.9)	40 (36.0)	24 (33.3)	13 (28.9)	17 (39.5)	4 (13.8)
Blood pressure/heart rate	116 (30.4)	51 (17.7)	39 (27.9)	21 (18.9)	25 (34.7)	8 (17.8)	10 (23.3)	7 (24.1)
Microbiota and metabolites	50 (13.1)	45 (15.6)	19 (13.6)	48 (43.2)	24 (33.3)	1 (2.2)	12 (27.9)	2 (6.9)
Hormonal parameters	52 (13.6)	49 (17.0)	34 (24.3)	8 (7.2)	6 (8.3)	10 (22.2)	10 (23.3)	2 (6.9)
Markers of liver function	35 (9.2)	33 (11.5)	17 (12.1)	16 (14.4)	3 (4.2)	7 (15.6)	2 (4.7)	4 (13.8)
Markers of kidney function	28 (7.3)	25 (8.7)	8 (5.7)	8 (7.2)	8 (11.1)	6 (13.3)	2 (4.7)	1 (3.4)
Genetic markers	24 (6.3)	20 (6.9)	13 (9.3)	8 (7.2)	6 (8.3)	2 (4.4)	5 (11.6)	1 (3.4)
Oxidative stress parameters	22 (5.8)	14 (4.9)	14 (10.0)	6 (5.4)	3 (4.2)	2 (4.4)	6 (14.0)	1 (3.4)
Minerals and vitamins	22 (5.8)	14 (4.9)	2 (1.4)	6 (5.4)	12 (16.7)	1 (2.2)	1 (2.3)	2 (6.9)
Cancer biomarkers	13 (3.4)	13 (4.5)	2 (1.4)	5 (4.5)	4 (5.6)	4 (8.9)	1 (2.3)	-
Hemochrome parameters	1 (0.3)	5 (1.7)	1 (0.7)	1 (0.9)	1 (1.4)	2 (4.4)	1 (2.3)	-
Markers of thyroid function	4 (1.0)	1 (0.3)	-	-	1 (1.4)	1 (2.2)	-	-
Other	9 (2.4)	10 (3.5)	1 (0.7)	-	1 (1.4)	1 (2.2)	-	-
**Funding sources**								
AMC/Hosp/US Govt/Other*	358 (94.0)	259 (89.9)	133 (95.0)	108 (97.3)	70 (97.2)	23 (51.1)	35 (81.4)	28 (96.6)
Industry	4 (1.0)	3 (1.1)	1 (0.7)	-	1 (1.4)	3 (6.7)	1 (2.3)	1 (3.4)
Both	19 (5.0)	26 (9.0)	6 (4.3)	3 (2.7)	1 (1.4)	19 (42.2)	7 (16.3)	-

*Data are reported as number of trials registered on ClinicalTrials.gov and percentage (%)*.

*AMC, Academic Medical Centers; Hosp, Hospitals; US Govt, United States Government*.

* * Other includes charities and foundations*.

**Figure 4:**
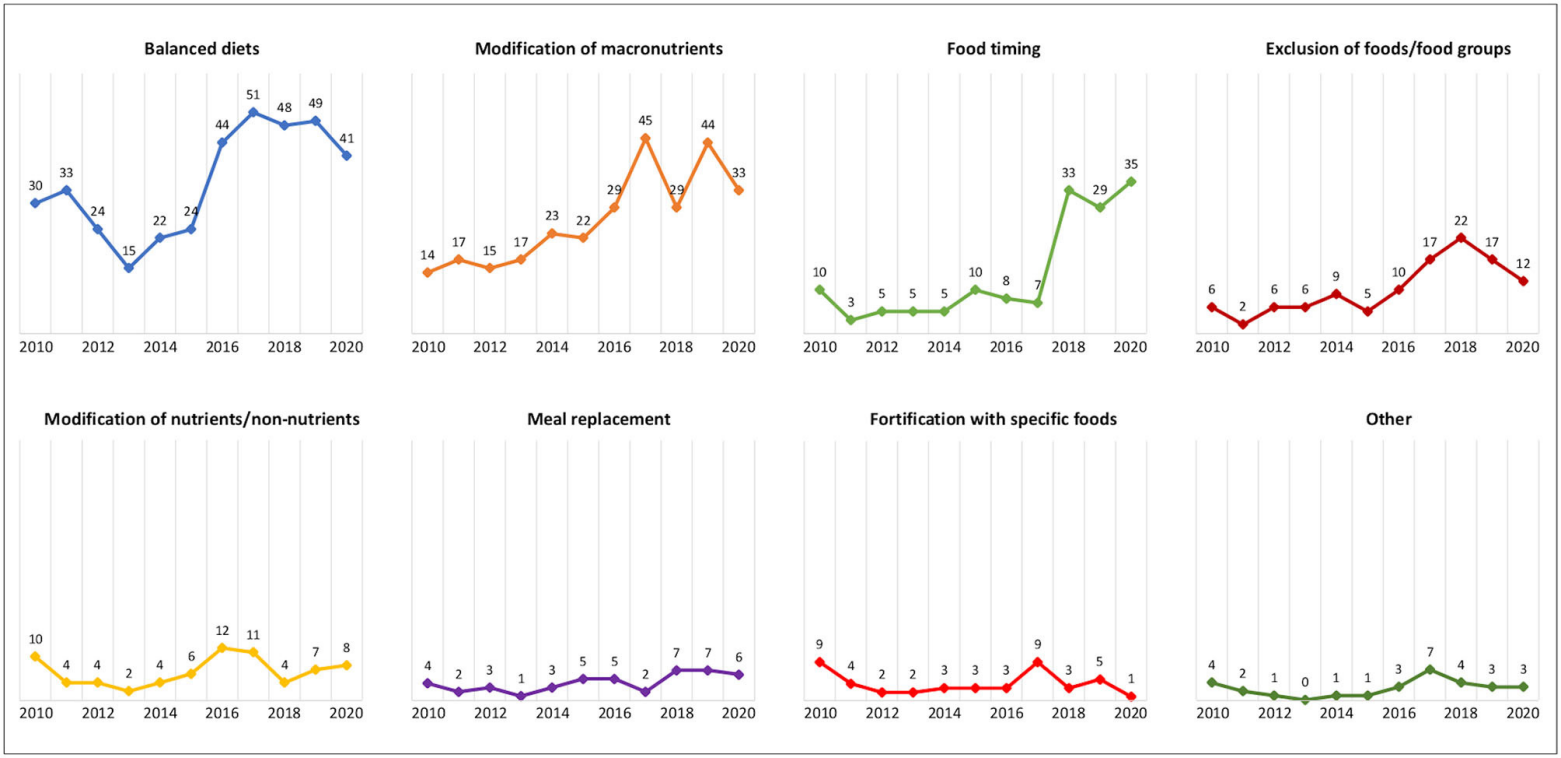
Number of registered trials over time according to dietary approaches.

Registration of trials over time according to dietary approaches is shown in Figure 4. While a downward trend has been observed for many dietary approaches, an increasing interest in balanced diets, diets based on the modification of macronutrients, and diets based on food timing has emerged since 2017.

## Discussion

To the best of our knowledge, this is the first study investigating the characteristics of clinical trials registered since 2010 and focused on the association between diet and health outcomes. The first intriguing data are related to the high number of studies included in the analysis, more than 1,000, among which, more than half were registered after 2017. This could be due both to an increased interest in deepening the relationship between diet and human health with *ad-hoc* clinical trials focused on different tasks of health outcomes and to the fact that in the last years, registration has been recommended also for dietary intervention studies. A major focus was on anthropometric measurements, which have been the most considered for all the diets, except the DASH one. This is likely due to the well-recognized role that an adequate dietary regimen has on the maintenance of an adequate body weight and, in turn, on the modulation of the glycemic and lipid profiles, as well as in the prevention of inflammatory status. Other than these “classic” clinical chemistry measurements, there is a growing interest in the study of the diet-derived gut microbiota modulation, both general and species-specific, which is more considered due to the increased awareness of its potential association with many health factors (13).

As to the type of diets investigated, balanced diets, comprising the Mediterranean diet, accounted for the largest proportion of trials registered on “ClinicalTrials.gov.” In this regard, it is worth to be noticed that most of these studies have been performed in Mediterranean countries, such as Italy and Spain. The second most investigated group of diets were those focused on the modification of macronutrients, among which, the ~85% were low-carbohydrate, ketogenic, high-protein, and low-fat diets. Despite there is no consensus on the percentage of energy coming from the three macronutrients in such diets, we can assume that all the regimens are roughly based on <45% of total energy from carbohydrates and/or >20% from proteins, with various ranges for energy from lipids (2). The increasing interest in these diets seems not to be country-related, being their study ubiquitously spread. More, researchers did not focus on a specific health outcome, despite the most recent theories focus on the putative impact on body weight and fat-lowering effect.

Another large group of diets present in the retrieved database belongs to the so-called “food timing” group, with the solely time-restricted feeding and intermittent-fasting diet studies accounting, together, for 90% registered trials. Despite some of such diets have long traditions, mainly sticking to religious purposes, an increasing interest has risen for the body effects of calorie restriction/fasting in certain times of the day or along one or more days of the week. A recent meta-analysis of clinical trials reported that such diets can promote lowering of the body weight and an amelioration of the fast glucose concentration but only in short-term studies (4–8 weeks, fasting time from 12 to 20 h) (14). Among the possible explications, the authors hypothesized that the calorie restriction at certain times of the day is able to boast the gluconeogenesis metabolism with amino acids and free fatty acids as substrates for energy supply, with a consequent glucose and insulin decreasing effect (14). In addition, the shift and/or decreasing of the daytime for meal consumption may have a role in restoring the circadian rhythms of the organism, affecting also the gut microbiota toward a more favorable composition leading to decreasing inflammatory and oxidative stress markers (15). Despite these promising presumptions and the increasing interest in associating “food timing” with healthy living and longevity (16), new studies are trying to investigate whether subjects are able to adhere and follow these regimens for longer times and without affecting their daily lifestyle habits.

Few studies (about 10% of the total) have been conducted on diets that excluded specific foods or food groups, such as gluten-free or vegetarian/vegan diets. The former ones are specifically designed for people with coeliac disease or gluten-sensitivity conditions, although they are often used with the aim of losing weight in a very tight time, likely due to the misperception that gluten may be responsible for weight gain and fastidious gut discomforts, such as bloating or soreness (17). Regarding vegetarian/vegan diets, these are the subject of growing interest given their potential effects not only on health markers (18) but also for being part of the so-called “sustainable dietary patterns,” caring for their environmental and ethical sustainability (19). However, the small number of studies suggests the need for further robust evidence to corroborate current findings and to better investigate the health impact of excluding some or all animal foods.

Besides focusing on the types of diets considered in the registered trials, it is noteworthy to analyze the main characteristics of such studies. Among them, duration is surely one of the most complicated aspects of the study design to be considered. From one side, the length of the study should consider the necessary period of time to expect a change in the considered outcomes, the fluctuation of such values along the time, etc. (20). However, other variables not linked to the biology of the organism may influence the choice of the duration: the adherence to the product/meal of the subject, by considering the possible drop-out for lacking adherence or the available funding to conduct the study for a longer period of time. This survey pointed out that most of the studies have a length of <12 weeks, despite a 12–25% of the studies reach also 24 weeks, which very often is not a sufficient frame time to have reliable information on the modulation of health outcomes by dietary interventions. Thus, an interesting finding of this survey is that more studies are needed to investigate the effect of diet on human health in the long term.

Regarding health status, on average only ~7% of the total registered trials enrolled apparently healthy individuals while, as expected, most of the studies enrolled overweight/obese subjects, followed by the ones affected by diabetes or other cardiovascular diseases. This is not surprising considering that it is difficult and, in many cases, not clinically relevant to the modulation of health outcomes in healthy people who have already health-related markers in physiological ranges. On the contrary, altered values of health or disease outcomes, i.e., body weight, glucose-, lipid- or inflammation-related markers in population with or at risk of chronic diseases, may be more sensitive to a dietary recommendation or to change of dietary regimens (21), such as the ones related to timing or diets based on the inclusion of specific foods.

Most of the registered trials have been performed by enrolling both men and women, which reflects the gold standard way to evaluate the health effects of foods/diet without gender/sex bias. However, it also emerged that there were a higher number of trials, up to 20% in the balanced diet group, conducted on women than those on men. These data may be discussed in different ways, mainly taking into account the main outcomes and the complexity of the two-body systems. In fact, an interesting US report pointed out that only some CVD trials focusing on hypertension, diabetes, and stroke, but not heart failure, coronary artery disease, and hyperlipidemia, showed a higher enrollment of women than men (22). In this survey, it can be hypothesized that studies focusing on body weight and anthropometric parameters, the first rated for number of trials, might have found more interest in women, who seem to care to their body size and aspects more than men (23). On the contrary, some studies excluding women might have considered some markers, which might be biased by the hormonal or metabolic differences between sex.

Regarding age, it is intriguing to notice that most of the studies were performed on adults or in both adults and older adults, while only 21 studies were focusing only on older adults (age > 65 years). Due to the well-recognized importance of dietary habits and lifestyle in healthy aging (24, 25), this represents an area worth to be explored, also with the final purpose to better define nutrition recommendations in older adults. However, not few drawbacks are present in the conduction of the study and in the caring of the subjects, i.e., presence of different illnesses, therapies with drugs not compatible with the studies, difficulties in furnishing meals, low adherence to the studies, etc. (26).

This work has several strengths and limitations worth to be noted. The first strength is related to the approach of using clinical trials registered in ClinicalTrials.gov, which allows to have a real-time picture of efforts carried out in these years, including those that have not led to publications, yet. Additionally, this approach allows to retrieve information that is often missing in publications, including details on the characteristics of the study population, the study design, and all outcome measures. Regarding limitations, it is noteworthy that the search on databases of registered clinical trials does not allow to retrieve information related to the results of the studies. Moreover, it cannot be excluded that the presence of other studies focused on diets and health that could have been found by using other specific terms, such as “eating” or “meal(s),” or that have not been registered or are registered in other databases. In fact, recent findings regarding the ICTRP revealed that less than 50% of the total clinical trial registrations belong to ClinicalTrial.gov database, with a decreasing trend due to a non-US clinical research rise of registrations (11). Further, we manually grouped diets into eight categories to facilitate data presentation, although this subjective categorization could have been performed also in other different ways. Lastly, we only focused our research on adults, missing the results of trials on subjects with <18 years old, mainly because of the very heterogeneous category of subjects, i.e., children, adolescents. This is explainable taking into consideration the very different dietary requirements for such categories of individuals and their restricted behaviors in following specific diets.

In conclusion, results from the present descriptive analysis underline the continuous interest not only in the study of traditional and balanced diets, such as the Mediterranean diet, but also for diets focused on modifications of macronutrients and, more recently, on food timing, with large variability in terms of duration of the study, selected outcomes, and characteristics of participants. These findings contribute to describe past efforts on this topic and to identify possible gaps and fields of research to cover in the next future to provide further evidence on the relation between diet and human health. Attributing specific health effects to diets is one of the greatest challenges in nutritional science. When clinical studies are underrepresented, as in some cases we have highlighted, a good strategy is to consider all available evidence and not just individual studies. Different studies, settings, and methodologies that produce similar results on the same question give a reasonably good indication of the existing relation between adherence to a particular diet and a specific health outcome.

## Data Availability Statement

Publicly available datasets were analyzed in this study. This data can be found here: https://clinicaltrials.gov/.

## Author Contributions

MD, DM, and DA conceived the study and were involved in the protocol design, data collection, analyses, and in the interpretation of the results. GP was involved in the protocol design and data collection. MD, CDB, DM, and DA drafted the manuscript. MP, PR, MS, and FS were involved in the interpretation of the results and reviewed the final draft. MD, DM, and DA had primary responsibility for the final content. All authors contributed to the article and approved the submitted version.

## Funding

DM is grateful for the support granted by Piano di sostegno alla ricerca-Linea 2, azione A, grant no. PSR2020-DMART.

## Conflict of Interest

The authors declare that the research was conducted in the absence of any commercial or financial relationships that could be construed as a potential conflict of interest.

## Publisher's Note

All claims expressed in this article are solely those of the authors and do not necessarily represent those of their affiliated organizations, or those of the publisher, the editors and the reviewers. Any product that may be evaluated in this article, or claim that may be made by its manufacturer, is not guaranteed or endorsed by the publisher.
